# Association between articular surface depression and functional outcomes after ORIF of tibial plateau fractures: a retrospective cohort study

**DOI:** 10.1007/s00068-025-03027-x

**Published:** 2025-12-11

**Authors:** Saad Madi, Said Chotta, Peter Abou Fadel, Mohamad Y Fares, Johannes Zeichen

**Affiliations:** 1 Johannes Wesling Klinikum Minden, Minden, Germany; 2 Rothman Orthopaedics, Philadelphia, USA

**Keywords:** Articular surface depression, ORIF, Patient-reported outcomes, Postoperative CT parameters, Tibial plateau fractures (TPF)

## Abstract

**Purpose:**

Tibial plateau fractures (TPF) involving the articular surface are among the most severe joint injuries of the lower extremities. The effect of precise reduction of TPF on postoperative outcomes remains uncertain. While several studies have emphasized the importance of restoring articular congruity, others have reported limited correlation between radiographic parameters and long-term function, leaving it unclear which aspects of reduction truly drive functional recovery. This study aims to evaluate and correlate postoperative CT scan parameters of patients who underwent ORIF for TPF with patient reported outcomes at 6 years post-surgery.

**Methods:**

A retrospective cohort study with cross-sectional outcome assessment was conducted on patients who underwent ORIF for tibial plateau fractures between 2016 and 2019. Patients without postoperative CT or lost to follow-up were excluded. Pre- and postoperative CT scans were analyzed for articular depression, plateau tilt, condylar widening, and posterior tibial slope. Patient-reported outcomes, including the Western Ontario and McMaster Universities Osteoarthritis Index (WOMAC) and the Knee injury and Osteoarthritis Outcome Score (KOOS), were obtained and correlated with CT findings to identify predictors of poor outcome.

**Results:**

Of 71 patients, 49 were included in the final analysis. The mean age was 51.6 years; 26 (53%) were men and 23 (47%) women. The mean follow-up was 6 years. The mean WOMAC and KOOS scores were 36.7 ± 19.1 and 58.9 ± 18.9, respectively. A post-operative articular step-off of ≥ 2.5 mm was significantly associated with worse WOMAC scores (*p* = 0.004). Other radiologic parameters, including condylar widening, tibial plateau tilt, and articular depression, showed no significant correlation with WOMAC or KOOS scores.

**Conclusion:**

A postoperative step-off deformity exceeding 2.5 mm on postoperative CT scans significantly impacts the mid-term functional outcome of patients undergoing ORIF after TPF, as evidenced by poorer WOMAC scores. This study emphasizes the importance of accurate TPF reduction to optimize patient outcomes.

## Introduction

Tibial plateau fractures (TPF) are common intra-articular fractures, accounting for 1–2% of all fractures and up to 8% of all fractures in the elderly population [1], Because they disrupt the load-bearing surface of the knee, TPFs are often associated with significant joint damage and functional impairment [2]. Treatment ranges from nonoperative management to minimally invasive approach to open reduction and internal fixation (ORIF) for complex patterns [3–6]. Surgical treatment aims at pain-free function through accurate joint and axis reconstruction, as non-anatomic reduction may cause pain, deformity, stiffness, and osteoarthritis, impairing function and quality of life [7–11]. Therefore, anatomical reconstruction with restoration of alignment is essential to prevent dysfunction and early arthritis [12, 13].

Performing postoperative Computer tomography (CT) scan is the most common method to evaluate the quality of reduction and joint congruency [14]. The radiological parameters defining failure of fixation after open reduction internal fixation (ORIF) in TPF are defined as follows: articular surface reduction (step-off) of >2–5 mm; valgus or varus angulation of >5–10°; posterior slope angulation of >5–10°, and condylar width widening of >5–10 mm [15–27]. Yet, the existing literature is inconsistent regarding the surgical indication for revision surgery of tibial plateau fractures, primarily when related to postoperative CT measurements. Frosch et al., in a certified educational article, emphasized the relevance of step-off deformity, recommending revision surgery for step-off >5 mm and even suggesting that values >2 mm should be considered unacceptable [15]. Parkkinen et al. similarly in his study on 73 patients with TPF reported that accurate reduction of articular step-off may help prevent post-traumatic osteoarthritis, although they found that it was not a reliable predictor of patient-reported outcomes, such as Western Ontario and McMaster Universities Osteoarthritis Index (WOMAC) and Lysholm scores, at five years [19]. However, both studies primarily addressed step-off deformity without analyzing additional postoperative radiological parameters. Extending beyond step-off, Shimizu et al. demonstrated that condylar widening was associated with lateral instability and an increased risk of secondary osteoarthritis, although no link was found between step-off and loss of reduction in their short one-year follow-up [28]. Thiagarajah et al. further underscored the importance of restoring tibial articular width to minimize radiographic osteoarthritis, though their use of Ilizarov fixation limits direct comparison with ORIF and excluded other parameters such as posterior slope or plateau tilt [29]. Taken together, these findings highlight contradictory evidence regarding the prognostic value of specific radiographic parameters, leaving the relationship between postoperative CT findings and functional outcome incompletely understood. To our knowledge, no study has systematically correlated multiple postoperative CT variables with mid-term functional outcomes. We therefore conducted a retrospective cohort study with cross-sectional outcome assessment to identify which postoperative CT parameters may predict mid-term clinical results as measured by the WOMAC and the Knee injury and Osteoarthritis Outcome Score (KOOS).

## Materials and methods

### Study population and characteristics

After obtaining approval from the Ethics Committee of the Medical Faculty, Ruhr-University Bochum, Germany (No. 2022 − 1010), we conducted a retrospective cohort study with cross-sectional outcome assessment of patients who sustained tibial plateau fractures at our institution, with a minimum follow-up of four years. A total of 71 consecutive patients treated at a single level-I trauma centre between January 2016 and December 2019 were enrolled, a timeframe chosen to ensure a minimum mid-term follow-up of 4–6 years; cases prior to 2016 were excluded due to incomplete digital records and lack of routine postoperative CT. Eligible patients were skeletally mature, had sustained an articular fracture of the tibial plateau, underwent pre- and postoperative CT imaging, and achieved at least four years of follow-up. While patients with isolated and associated injuries were eligible, no cases with severe concomitant injuries likely to impair long-term mobility were present in this study. Patients with rheumatic or neurological diseases, periprosthetic fractures, or missing postoperative CT scans were excluded. Additional exclusion criteria for the cross-sectional outcome assessment (KOOS and WOMAC) were loss to follow-up, inability to be contacted, relocation abroad or death. Demographic and clinical data, including age, sex, and comorbidities such as osteoporosis, were recorded at the time of hospital admission.

### Classification and operative management

**Fig. 1:**
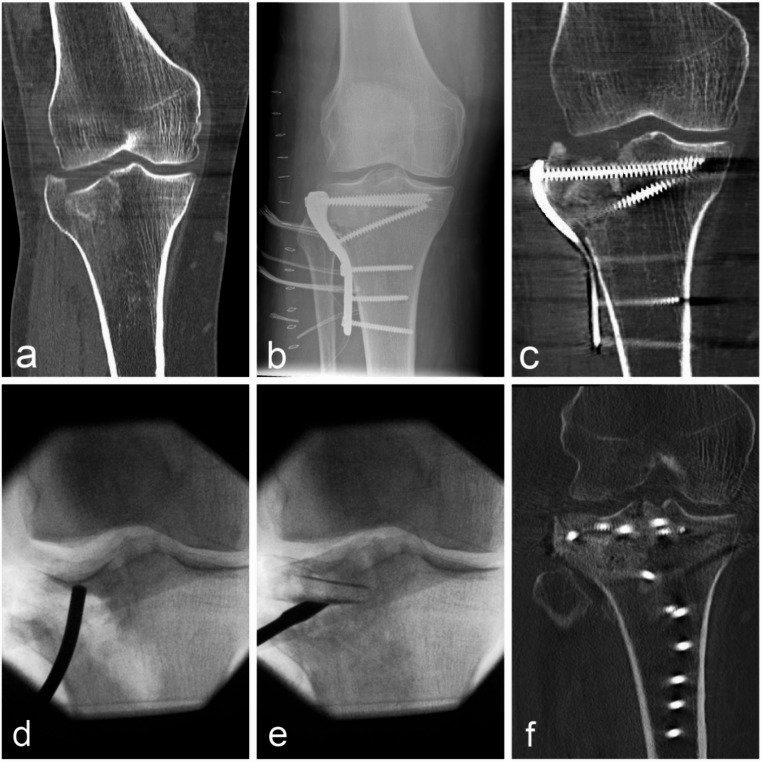
(**a**) Pre-operative CT scan of a 57-year-old male patient who was kicked by a bull and sustained a proximal tibia fracture type II according to the Schatzker classification. (**b**) The initial fracture was treated with open reduction and internal fixation using a lateral locking plate at another institution. However, the patient subsequently presented to our center with persistent stabbing pain and discomfort that had not improved despite pain management and physical therapy. (**c**) CT scans performed at our center revealed a depression of the lateral tibial plateau by 5.3 mm, primarily in the dorsal region. (**d**) Four weeks later, revision surgery was undertaken. The initial fixation material was removed, and an arthroscopically assisted reconstruction of the articular surface was performed. The entire dorsolateral quadrant was elevated by 5 mm. (**e**) The defect was then filled with both an allogenic tricortical bone chip and allogenic spongiosa. (**f**) Postoperatively, the tibial plateau was restored to its physiological height, resulting in a smooth reconstruction of the articular surface. The patient experienced complete relief from the stabbing pain and was pain-free following the procedure

Preoperative CT was performed on all patients to assess fracture morphology and guide surgical planning. In selected cases, particularly in the context of high-energy trauma, complex fracture patterns, or when severe or clinically suspected soft-tissue injuries (e.g., meniscal or ligamentous lesions) were present, MRI was additionally obtained at the discretion of the treating surgeon. Depending on fracture morphology and surgeon preference, fixation was performed using implants from Synthes (DePuy Synthes, Zuchwil, Switzerland) or Intercus (Intercus GmbH, Rudolstadt, Germany). Among the 21 patients with type II fractures (43%), a lateral column buttress plate was used via a standard lateral or anterolateral approach to prevent secondary valgus deformity. Fourteen patients (29%) had type IV fractures, which were managed with a medial column buttress plate to prevent varus deformity. Ten patients (20%) had type V fractures and four patients (8%) had type VI fractures; these were treated with a double-plate system depending on the individual fracture morphology. In two of the type V cases, the posterior column was involved, and an additional posterior plate was applied to support the shear fragment and prevent displacement. Depressed fragments of the joint surface were elevated under visual control. The bone defect was filled with Allograft cancellous chips (DIZG GmbH, Berlin, Germany), when needed, to support the elevated fragments. To avoid residual step-off deformities, we adopted an open arthroscopic technique that involves inserting a 2.8 mm arthroscope directly through the open surgical approach without additional arthroscopic portals or water pressure. Patients were treated with a hinged knee brace permitting early passive range of motion. Weight-bearing was limited to non-weight bearing or partial weight bearing of up to 10 kg for 8–12 weeks, depending on intraoperative findings, fracture complexity, and surgeon preference. In the case of critical soft tissue conditions that preclude early definitive fixation, and by open fractures, it is our general practice to use temporary external fixation. Two orthopaedic surgeons with more than 20 years of surgical experience performed all surgeries. The imaging findings and surgical intervention of one selected case are illustrated in Fig. 1.

### CT scan measurements and study parameters

Postoperative CT scans were performed for all patients during the inpatient stay at the hospital. Criteria for TPF mal-reduction were measured on the postoperative CT scans, including articular surface depression (step-off), plateau tilt, condylar widening, and posterior tibial slope. Articular surface depression was measured in the coronal plane as the vertical distance between the point of maximum depression and the joint line. Previous studies have shown that a step-off >2.5 mm is associated with poor clinical outcomes, therefore, this threshold was adopted as a criterion for mal-reduction [26, 30]. Since we did not have a contralateral CT scan of the uninjured knee, we could not calculate condylar widening as the difference between the healthy and the fractured sides in the axial view. Therefore, we measured condylar widening as the horizontal distance from the point of maximum fracture fragment, with a threshold of >5 mm [19]. Tibial plateau tilt was measured in the coronal plane and defined as the angle between the surface of the tibial plateau and the longitudinal axis of the tibia, with a valgus or varus angulation of >5° deemed unsatisfactory [19]. Posterior tibial slope was measured in the sagittal plane and defined as the angle created by the tibial plateau and the long axis of the tibia [31]; a slope >10° was deemed unsatisfactory.

### Clinical outcomes

For the cross-sectional outcome assessment, patients were re-contacted and provided written informed consent. They subsequently completed standardized questionnaires: WOMAC, which assesses pain, stiffness, and physical function [32], and KOOS, which evaluates pain, symptoms, activities of daily living, sport and recreation, and knee-related quality of life [33]. These were compared with postoperative CT scan findings to explore predictors of patient dissatisfaction or suboptimal outcomes. Medical records were reviewed, noting the treatment course, postoperative CT measurements, complications, and any revision procedures.

### Statistical analyses

Statistical analyses were performed using Stata Statistical Software, Release 14.1 (StataCorp LLC, College Station, TX). Categorical variables were expressed as percentages, and continuous variables as mean and standard deviation. Univariate analysis of each variable was performed using a 2-tailed Student t-test for continuous variables and Fisher’s exact test for categorical variables. Normality of continuous variables was assessed using the Shapiro–Wilk test. Normally distributed data were reported as means with standard deviations and analyzed using parametric tests. Non-normally distributed data were reported with medians and interquartile ranges. For key outcomes that were non-normally distributed (e.g., WOMAC), nonparametric tests (Wilcoxon rank-sum for independent groups or Wilcoxon signed-rank for paired comparisons) were performed as sensitivity analyses to confirm the robustness of parametric results. The significance level was set at *p* < 0.05.

## Results

### Patient demographics and characteristics

Of the 71 patients, 49 patients (69%) were included in our final cohort. A total of 22 patients were excluded: 4 patients had no postoperative CT scan, 3 patients sustained a periprosthetic fracture, 5 patients died, and 10 patients were lost to follow-up. The mean age of the included patients was 51.6 years (Standard deviation [SD] = 15.5; Range, 18–87), with 26 males (53%) and 23 females (47%). The mean follow-up was 5 years and 11 months (SD 1.23; Range, 4.33–8.17). According to the Schatzker classification, 21 cases were type II (43%), followed by type IV in 14 cases (29%), type V in 10 cases (20%), and type VI in 4 cases (8%). A single-plate system was used in 27 patients (55%), and a multiple-plate system was used in 22 patients (45%).

A total of 12 patients (29%) sustained concomitant soft tissue injuries. These included six meniscal tears (five isolated lateral and one combined medial and lateral), three anterior cruciate ligament (ACL) ruptures (one isolated, one with an associated medial collateral ligament (MCL) rupture, and one with a concomitant lateral meniscus tear), two isolated MCL ruptures, one lateral collateral ligament (LCL) rupture, one posterior cruciate ligament (PCL) rupture, and one patellar tendon rupture. All soft tissue injuries were identified preoperatively, in case an MRI was performed or diagnosed intraoperatively. Two patients who sustained an open fracture (Gustilo grade II) were treated with a staged protocol with urgent irrigation, debridement, external fixation, antibiotic prophylaxis, and delayed definitive treatment. In addition, five patients (11%) had manifest osteoporosis. Table 1 describes the overall patient demographics and characteristics.

**Table 1:** Patient demographics and characteristics. SD, standard deviation; ACL, anterior cruciate ligament; PCL, posterior cruciate ligament. Percentages are based on the total cohort (*n* = 49). Note that some patients sustained more than one concomitant soft tissue injury

Variable	No. of patients (%)
Age (Mean ± SD)	51.6 ± 15.5
Sex	
Male	26 (53%)
Female	23 (47%)
Schatzker classification	
Type II	21 (43%)
Type IV	14 (29%)
Type V	10 (20%)
Type VI	4 (8%)
Implant System	
Single-plate	27 (55%)
Multiple-plate	22 (45%)
Soft tissue injury	
Meniscal injury	6 (12%)
ACL Injury	3 (6%)
PCL Injury	1 (2%)
Patellar tendon rupture	1 (2%)
LCL Injury	1 (2%)
MCL Injury	2 (4%)
Open Fracture	3 (6%)
Osteoporosis	5 (10%)

### Postoperative CT scan parameters

Postoperative evaluation showed that 35 patients (71%) had an articular step-off < 2.5 mm, while 14 patients (29%) had a step-off ≥ 2.5 mm. In the coronal plane, 30 patients (61%) demonstrated a tibial plateau tilt < 5°, whereas 19 patients (39%) had a tilt ≥ 5°. In the sagittal plane, 12 patients (25%) had a posterior tibial slope < 10°, while 37 patients (75%) showed a slope ≥ 10°. Assessment of condylar widening in the axial plane revealed 43 patients (88%) with widening < 5 mm and 6 patients (12%) with widening ≥ 5 mm.

### Clinical outcomes

The mean WOMAC score was 36.7 (SD 19.1; range, 0–64). WOMAC scores varied significantly by Schatzker classification (*p* = 0.013): 31.8 in type II, 30.9 in type IV, 46.5 in type V, and 57.8 in type VI. Mean WOMAC scores differed significantly between patients with a step-off < 2.5 mm and those with a step-off ≥ 2.5 mm (32.1 ± 3.1 vs. 47.9 ± 4.3, *p* = 0.004). There was no significant difference in WOMAC score amongst different variables (Table 2). The mean KOOS score was 58.9 (SD 18.9; Range, 21–100). The Mean KOOS score also varied significantly by Schatzker classification (*p* = 0.010): 59.0 in Type II, 66.7 in Type IV, 58.3 in Type V, and 18.1 in Type VI. There was no significant difference in the overall KOOS score or the KOOS sub-categories in patients with varying CT measurements (Table 3).

**Table 2:** Predictors for WOMAC outcome scores

Variable	No. of patients (%)	WOMAC score				*P*-value
		Mean	SD	Median	IQR	
Sex						0.154
Male	26 (53%)	34.0	4.2	36	16–55	
Female	23 (47%)	39.7	3.4	41	31–52	
Schatzker classification						0.013*
Type II	21 (43%)	31.8	19.6	31	16–48	
Type IV	14 (29%)	30.9	17.6	35	19–41	
Type V	10 (20%)	46.5	15.4	48	39–58	
Type VI	4 (8%)	57.8	3.6	56.5	55.5–60	
Step-off						0.004*
Satisfactory (< 2.5 mm)	35 (71%)	32.1	3.1	33	16–48	
Unsatisfactory (≥ 2.5 mm)	14 (29%)	47.9	4.3	54	38–58	
Tibial Plateau Tilt						0.265
Satisfactory (< 5°)	30 (61%)	35.3	3.4	35.5	19–50	
Unsatisfactory (≥ 5°)	19 (39%)	38.8	4.7	43	31–55	
Posterior Tibial Slope						0.549
Satisfactory (< 10°)	12 (24%)	37.3	4.8	35.5	28–51.5	
Unsatisfactory (≥ 10°)	37 (76%)	36.5	3.3	41	19–55	
Condylar widening						0.061
Satisfactory (< 5 mm)	43 (88%)	35.1	2.8	38	19–52	
Unsatisfactory (≥ 5 mm)	6 (12%)	48.0	8.4	56.5	37–63	

**Table 3:** Predictors for KOOS outcome scores

Variable	No. of patients (%)	KOOS score				*P*-value
		Mean	SD	Median	IQR	
Sex						0.705
Male	24 (55%)	60.3	4.2	56.5	49–79.5	
Female	20 (45%)	57.2	3.9	59.5	45–67.5	
Schatzker classification						0.010*
Type II	17 (43%)	59.0	18.8	59	48–79	
Type IV	14 (29%)	66.7	18.0	63.5	53–80	
Type V	9 (20%)	58.3	10.6	57	55–63	
Type VI	4 (8%)	32.0	18.1	24	21.5–42.5	
Step-off						0.903
Satisfactory (< 2.5 mm)	32 (76%)	61.2	3.4	60.5	51–78	
Unsatisfactory (≥ 2.5 mm)	12 (24%)	52.8	5.2	52.5	37.5–63.5	
Tibial Plateau Tilt						0.472
Satisfactory (< 5°)	27 (61%)	58.7	3.7	59	49–77	
Unsatisfactory (≥ 5°)	17 (39%)	59.1	4.6	56	48–66	
Posterior Tibial Slope						0.309
Satisfactory (< 10°)	12 (27%)	56.5	5.1	60.5	41–68	
Unsatisfactory (≥ 10°)	32 (73%)	59.8	3.5	58	49.5–77.5	
Condylar widening						0.998
Satisfactory (< 5 mm)	38 (86%)	62.7	2.7	60.5	52–78	
Unsatisfactory (≥ 5 mm)	6 (14%)	34.7	5.9	30.5	22–48	

### Complications and reoperations

In the overall cohort, six patients (12%) experienced surgical complications. Three patients developed postoperative wound infections: two occurred within the first four weeks and were successfully managed with surgical wound debridement and aggressive irrigation without implant removal, while one late infection (at 3 Months) required implant removal. At the time of removal, the fracture had achieved sufficient consolidation to allow protected non-weight bearing until complete healing. Two patients developed arthrofibrosis with persistent loss of motion unresponsive to physiotherapy, necessitating surgical arthrolysis. One patient sustained a patellar tendon rupture requiring surgical repair three months postoperatively. Additionally, three patients presented with compartment syndrome on admission and underwent immediate revascularization with prophylactic four-compartment fasciotomy of the lower extremity. As these cases required delayed ORIF, they were not considered postoperative complications.

## Discussion

In this study, we analyzed the relationship between postoperative CT parameters and mid-term functional outcomes after ORIF of tibial plateau fractures, with a mean follow-up of six years. Of the evaluated parameters, only an articular step-off ≥ 2.5 mm was significantly correlated with poorer WOMAC scores, underscoring the particular importance of restoring joint congruity. In contrast, KOOS scores did not differ significantly across the assessed radiographic measures.

Residual articular depression significantly influenced mid-term outcomes in our cohort, with even small variations in joint congruity translating into meaningful differences in knee function and patient satisfaction at six years.

Several studies have suggested that precise joint reduction is not essential for good clinical outcomes, with some long-term series even reporting no correlation between residual depression (up to 10 mm) and function or osteoarthritis, while factors such as knee stability appeared to play a more decisive role [14, 24, 34–39]. However, a growing body of evidence indicates the opposite, suggesting that residual steps or gaps greater than 2 mm are associated with worse outcomes and an increased risk of secondary osteoarthritis [11, 15, 19]. Singleton et al. reported that smaller residual depression was associated with better functional outcomes at 12 months [26], findings consistent with our results. Importantly, in contrast to earlier radiographic studies, both our study and Singleton’s relied on CT-based assessment, providing a more accurate evaluation of post operative articular surface restoration.

In our study, 76% of patients had a postoperative posterior tibial slope (PTS) above 10°, but this did not translate into poorer mid-term outcomes. This contrasts with reports stressing the need for anatomical slope restoration to maintain stability and ligament balance [40, 41]. A likely explanation is methodological: most previous studies relied on plain radiographs, which, as Gwinner et al. highlighted, lateral radiographs have limited accuracy because they cannot reliably distinguish between the medial and lateral compartments [42]. Our CT-based measurements are more sensitive and yield higher values, which may explain the discrepancy.

Insufficient intraarticular visualization of the fracture is a major cause of surgical failure [43]. Standard two-dimentional fluoroscopy misses nearly 60% of intra-articular fractures and only detects step-offs greater than 5 mm [38, 39, 44] To address this, we used fracturoscopy as described by Krause et al., which allows direct assessment of residual steps and gaps in the posterior plateau; unlike conventional arthroscopy, this technique avoids fluid extravasation and reduces postoperative swelling or compartment syndrome risk [15, 44, 45]. In our series, fracturoscopy was sufficient to evaluate reduction without the need for additional arthrotomies.

Moreover, attaining postoperative CT scans following ORIF for TPF can provide a detailed assessment of joint congruency. In a study by Hackl et al., intra-articular analysis of TPF determined from radiographs had to be corrected in 40% of all cases after CT was performed [46]. In our study, assessment of joint congruency would not have been possible without precisely measuring the reduction in the coronal, axial, and sagittal planes on the postoperative CT images. Therefore, we also emphasize that CT scans are invaluable for analysing postoperative reduction and cannot be replaced by plain radiographs.

Although a statistically significant association between WOMAC and step-off deformity was found, but not with KOOS, we believe this difference can be attributed to the different focus of the two questionnaires [33, 47]. The WOMAC, developed specifically for the assessment of osteoarthritis, focuses on pain, stiffness, and physical function, which are variables that are more likely to be influenced by the structural impact of step-off deformity [47]. This specificity enables WOMAC to detect subtle limitations directly related to deformities. In contrast, the KOOS is designed for a broader, often younger, and more active population that does not correspond to the average age of our study population and includes additional dimensions, such as sports and leisure [33, 48]. In our study, the limited sample sizes within the Schatzker type IV–VI subgroups restrict the statistical power of the analysis; therefore, the KOOS and WOMAC outcomes in these subgroups should be regarded as descriptive and interpreted with caution. Moreover, the wide variability in WOMAC and KOOS scores further reflects the heterogeneity of our cohort, including differences in fracture type, fixation strategy, and concomitant soft-tissue injuries.

This study has several limitations. First, the cohort size of 49 patients is relatively small, which limits the generalisability of our findings and may constrain external validity and statistical power, although the mean follow-up of 6 years represents a strength. Second, the retrospective design carries inherent risks of bias, including potential treatment bias, as surgical decisions were made at the discretion of the treating surgeons. third, the study relied only on subjective questionnaires (WOMAC and KOOS), as clinical and radiographic data were not available at the final follow-up. This prevented assessment of objective parameters such as range of motion, gait, radiographic healing, and post-traumatic osteoarthritis. Fourth, the small number of patients with concomitant soft-tissue injuries limits conclusions about their impact on outcomes, although such cases reflect real-world trauma practice. Similarly, adjustment for comorbidities such as osteoporosis was not feasible due to the low number of affected patients. In addition, pre- and postoperative leg alignment (varus/valgus) could not be evaluated because full-leg radiographs were not obtained, which may also have influenced the outcomes. Fifth, only univariate analyses were performed; multivariate regression was not feasible due to limited sample size across heterogeneous fracture types. Finally, the wide variation in WOMAC and KOOS scores likely reflects the heterogeneity of our cohort, which included a spectrum of fracture patterns (Schatzker II–VI) and various fixation strategies (single, double, and posterior plating), as well as concomitant soft-tissue injuries. We acknowledge that outcomes may differ substantially by fracture subtype and fixation method, but the limited sample size precluded meaningful subgroup analysis. In addition, a proportion of patients were lost to follow-up, which may have biased the results.

## Conclusion

Residual articular depression greater than 2.5 mm on postoperative CT was significantly associated with worse mid-term functional outcomes following ORIF of tibial plateau fractures, as measured by the WOMAC score at a mean follow-up of 6 years. These findings underline the importance of accurate articular reduction for long-term knee function and patient satisfaction. Future studies with larger cohorts and systematic CT-based analyses are warranted to assess the influence of postoperative parameters on long-term clinical, functional, and radiological outcomes.

## Data Availability

No datasets were generated or analysed during the current study.
